# *Eucalyptus obliqua* tall forest in cool, temperate Tasmania becomes a carbon source during a protracted warm spell in November 2017

**DOI:** 10.1038/s41598-022-06674-x

**Published:** 2022-02-17

**Authors:** Timothy J. Wardlaw

**Affiliations:** ARC Training Centre for Forest Values, University of Tasmania, Hobart, Australia

**Keywords:** Plant sciences, Climate sciences, Ecology

## Abstract

Tasmania experienced a protracted warm spell in November 2017. Temperatures were lower than those usually characterising heatwaves. Nonetheless the warm spell represented an extreme anomaly based on the historical local climate. Eddy covariance measurements of fluxes in a *Eucalyptus obliqua* tall forest at Warra, southern Tasmania during the warm spell were compared with measurements in the same period of the previous year when temperatures were closer to average. Compared with previous year, the warm spell resulted in 31% lower gross primary productivity (GPP), 58% higher ecosystem respiration (ER) and the forest switching from a carbon sink to a source. Significantly higher net radiation received during the warm spell was dissipated by increased latent heat flux, while canopy conductance was comparable with the previous year. Stomatal regulation to limit water loss was therefore unlikely as the reason for the lower GPP during the warm spell. Temperatures during the warm spell were supra-optimal for GPP for 75% of the daylight hours. The decline in GPP at Warra during the warm spell was therefore most likely due to temperatures exceeding the optimum for GPP. All else being equal, these forests will be weaker carbon sinks if, as predicted, warming events become more common.

## Introduction

The capacity for many forests to take-up carbon is diminished during heatwaves^1–3^. In most reported cases, this diminution in carbon uptake is the consequence of the need to regulate stomatal conductance to reduce water loss under drought conditions or high evaporative demand^2–4^. However, there are differences among forest ecosystems in their response to heatwaves^5^. During one heatwave event in Australia for example, van Gorsel, et al.^3^ found the pattern of responses in carbon and energy fluxes in eucalypt dry sclerophyll forests and woodlands across southern Australia differed from that of a tall eucalypt forest in south-eastern Australia. In water-limited dry sclerophyll forests and woodlands, carbon uptake declined in concert with decreased evapotranspiration, as evidenced by latent heat flux declining relative to sensible heat flux. The reverse occurred in the energy-limited tall eucalypt forest during the heatwave—carbon uptake increased, and evapotranspiration remained high as evidenced by latent heat flux increasing relative to sensible heat flux.

Patterns of carbon and energy fluxes during the heatwave event reported by van Gorsel, et al.^3^ were consistent with photosynthesis and transpiration being coupled through mechanisms of stomatal controls articulated in^6^ that regulate water loss and maintenance of intercellular CO_2_ concentrations. However, measurements by Drake et al.^7^ on young *Eucalyptus paramattensis* trees housed within whole-tree growth-chambers, suggested photosynthesis and transpiration could become decoupled during extreme heatwave conditions (maximum air temperatures of 43–44 °C over four days). De Kauwe, et al.^8^ were unable to show conclusively that carbon and energy fluxes decoupled in forest ecosystems experiencing extreme heatwaves (consecutive days with maximum temperatures > 35 °C). One of the reasons the authors proposed for this was the possibility that atmospheric demand associated with high vapour pressure deficits (VPD) during extreme heatwaves could cause the measured increased transpiration even with decreased stomatal conductance.

The study of De Kauwe, et al.^8^ excluded temperate forest sites in south-eastern Australia because they rarely met or exceeded the threshold temperatures used as their heatwave definition. Perkins and Alexander^9^ argue against simply describing extreme heatwave events in terms of consecutive days above a threshold temperature because they do not properly account for the regional differences in historical climates. Instead, they advocate regionally adjusted metrics such as consecutive days that exceed the 90th percentile value for the calendar day. Such regionally adjusted metrics should also better account for any local adaptation by the forest to the regional climate.

Optimum temperature for the gross primary productivity (GPP) of a forest has been shown to vary according to the historical climate of the forest site^10–12^. When the thermal optimum for GPP is exceeded, the net carbon balance of the forest rapidly declines because GPP decreases in concert with increasing ecosystem respiration^13^. In Australia, the most productive forest ecosystems occur in high rainfall areas at either end of a temperature gradient^14^. On mesic sites in the cool, temperate regions of south-eastern Australia, high forest productivity coincides with low temperature optimum for GPP^12^. While van Gorsel, et al.^3^ detected no decline in GPP during a heatwave event in 2012–2013 in one temperate eucalypt forest on a mesic site, it was the only such forest included in that study. There is a need for more observations of the GPP response to heatwaves of eucalypt forests growing on mesic sites.

The entire state of Tasmania experienced record-breaking heatwave in November 2017^15^. The Warra Supersite^14,16^, part of Australia’s Terrestrial Ecosystem Research Network, and situated near the southern extent of the tall eucalypt forests in Australia, became operational in March 2013. Meteorological and micro-meteorological instruments installed on an 80-m tower above a *E. obliqua* tall forest at the Warra Supersite, monitored carbon, water and energy fluxes during the 2017 heatwave. Here I report the measured response of a *E. obliqua* tall forest in southern Tasmania to the 2017 heatwave and compare it with those previously reported for heatwaves in other temperate eucalypt forests.

## Results

### Analysis of historical heatwaves in southern Tasmania

**Figure 1:**
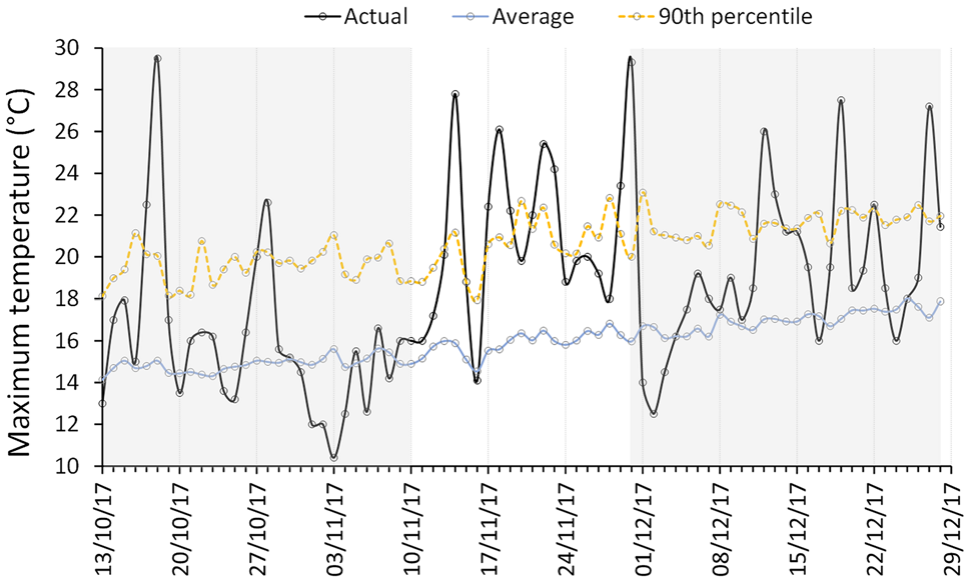
Daily maximum temperatures recorded at Cape Bruny Lighthouse during the 2017 warm spell (10–30th November) and the month either side (shaded). Average daily maximum temperatures during the period are plotted together with the average and 90th percentile values for those days calculated from long-term (1923–2020) records.

Over nearly a century of measurements, 99 in-growing-season (Austral spring–summer) heatwave events have been recorded at Cape Bruny Lighthouse (59 km southeast of Warra Supersite). Two of those events occurred in November 2017: 17th–19th and 21st–23rd. Those two events were embedded within a protracted warm period extending from 10th to 30th November during which all but one day exceeded the average daily maximum temperature and ten days had maximum temperatures that matched or exceeded the 90th percentile values (Fig. 1) for their calendar days. The departure from average temperatures during the 21-day warm spell in November 2017 was the largest on record (z-score = 3.86, equating approximately to a 1 in 800 year event). Despite this, there were no days where maximum temperature exceeded 30 °C: more than half of the 99 heatwave events recorded at Cape Bruny Lighthouse were hotter than the two November 2017 events. Hereafter, the November 2017 warm spell with embedded heatwaves is referred to simply as the “warm spell”. Temperatures leading up to the warm spell and in the week following the warm spell were much cooler, with many days below the long-term average (Fig. 1).

The average daily maximum temperature during the same period (10–30 November) in 2016 was a much milder 16.6 °C. While slightly above the long-term average (1923–2020) of 15.9 °C for this period at the Cape Bruny Lighthouse, the maximum temperatures in 2016 were not significantly different from the long-term average (z-score = 0.55). Accordingly, 10–30 November 2016 was used to represent baseline conditions and was referred to as the “comparison period”.

### Weather conditions at Warra Supersite during the warm spell

**Figure 2:**
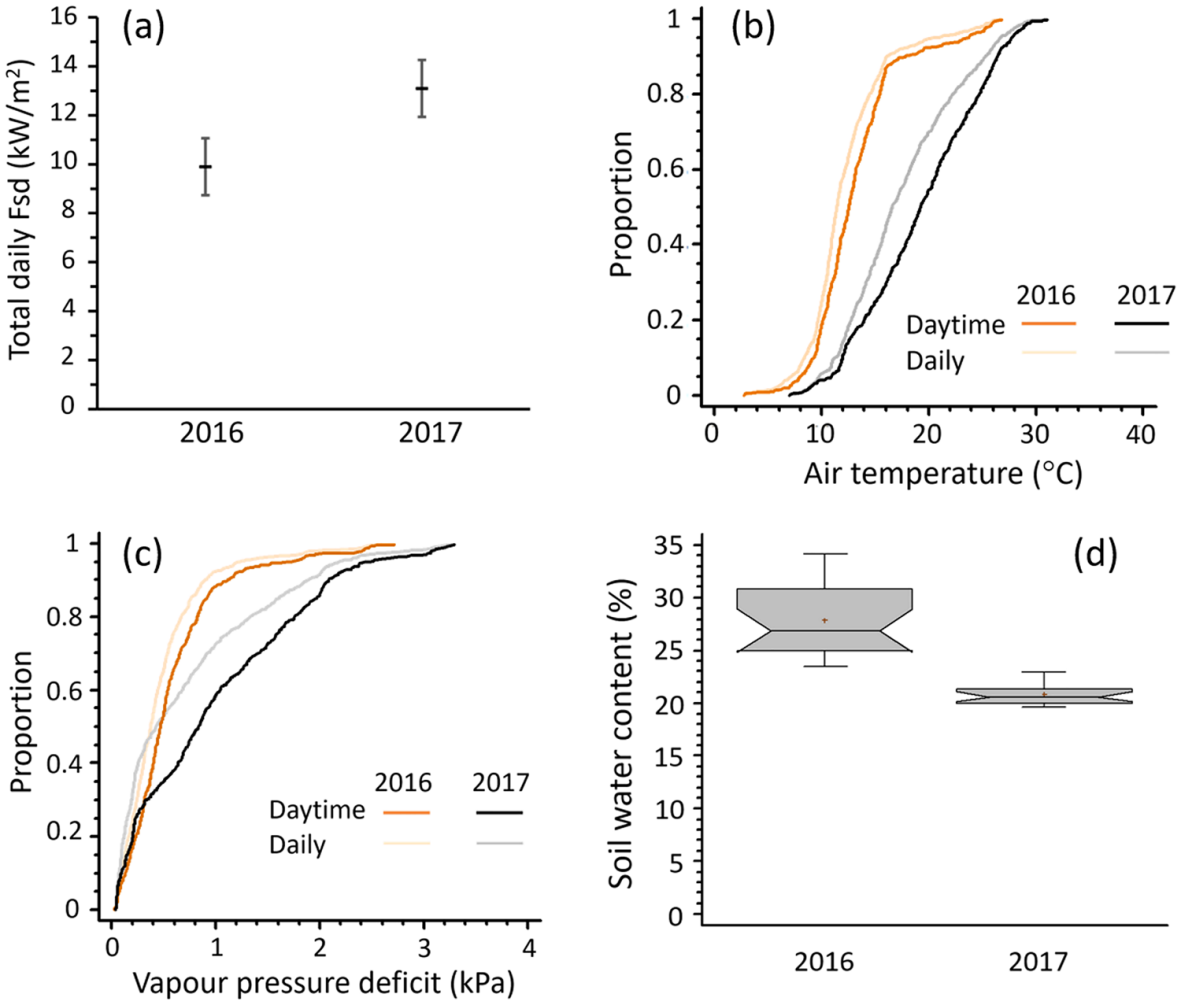
Climate conditions at Warra Supersite during the 2017 warm spell and the 2016 comparison period. Plots of daily averages or sums for the period (10–30 November), are shown for: (**a**) average total daily incoming shortwave radiation (with 95% least significant difference error bars); quantile plots of 30-min averages of (**b**) air temperature and (**c**) vapour pressure deficit; and (**d**) box and whisker plot of average daily soil water content.

The forest received significantly more incoming shortwave radiation (Fsd) during the warm spell (F_1,40_ = 8.12; MSE = 13.2; *P* = 0.007), equating to a 32% increase in Fsd compared with comparison period (Fig. 2a). Unsurprisingly, it was significantly warmer during the warm spell (F_1,40_ = 46.1; MSE = 5.9; *P* < 0.001). More than 75% of the 30-min daytime temperature records during the warm spell exceeded 15 °C, whereas only about 24% exceeded 15 °C in the comparison period (Fig. 2b). Despite the general warming in the warm spell, only six 30-min periods had air temperature of 30 °C or more. In parallel with temperature, vapour pressure deficit (VPD) was significantly higher during the warm spell than the comparison period (F_1,40_ = 6.3; MSE = 0.26; *P* = 0.017 for log-transformed data). Despite being higher during the warm spell than the comparison period, VPDs were still relatively low—only sixteen 30-min periods during the warm spell had a VPD of 3 kPa or more (Fig. 2c). The soils at Warra were significantly drier during the warm spell (Kruskal–Wallis statistic = 30.8; *P* < 0.001) than the comparison period (Fig. 2d) but nonetheless did not drop below 20% water content.

Examination of the antecedent climate in the 10 weeks prior (September 1–November 9) to the warm spell found Fsd and VPD conditions were comparable with the same period in 2016. Daytime air temperatures in 2017 were slightly, but significantly, lower (Kruskal–Wallis statistic = 5.98; *P* = 0.014) and had significantly more extreme values (Kolmogorov–Smirnov statistic = 2.26; *P* < 0.001) than in 2016. Soils were significantly drier in 2017 than 2016 (Kruskal–Wallis statistic = 30.8; *P* < 0.001), but again did not drop below 20%. Subsequent climate conditions in the month after the warm spell (1–31 December) had Fsd, daytime temperature and VPD measurements that were comparable with the month after the comparison period. Soil moisture remained significantly lower in the month after the warm spell than the month after comparison period (Kruskal–Wallis = 1362; *P* < 0.001).

### Turbulent fluxes at Warra Supersite during the warm spell

Energy balance closure tests how well the requirement for conservation of energy has been satisfied by energy flux measurements at the site, and by extension turbulent fluxes overall. Closure of energy balance requires the available energy (net radiation (Fn) less the energy absorbed by, and lost from, the ground -the ground heat flux (Fg)) is fully dissipated through latent heat, (Fe), the energy involved in evaporating water (including by transpiration) and sensible heat, (Fh), the energy involved in heating air. This was tested by regressing the sum of Fe and Fh against the available energy, Fn–Fg. Full closure of the energy balance is reflected by a linear regression that has a slope of 1 and a y-axis intercept of 0. The slopes of the regressions of Fe + Fh versus Fn-Fg (after forcing the y-axis intercept to 0) during the warm spell and the comparison period were 0.68 (R^2^ = 0.91) and 0.66 (R^2^ = 0.90), respectively. While 66–68% closure is less than ideal, the energy balance closure was comparable between the two periods. Energy balance closure increased to 74% for the warm spell and 69% for the comparison period when energy storage in aboveground biomass was accounted for according to the method of^17^.

**Figure 3:**
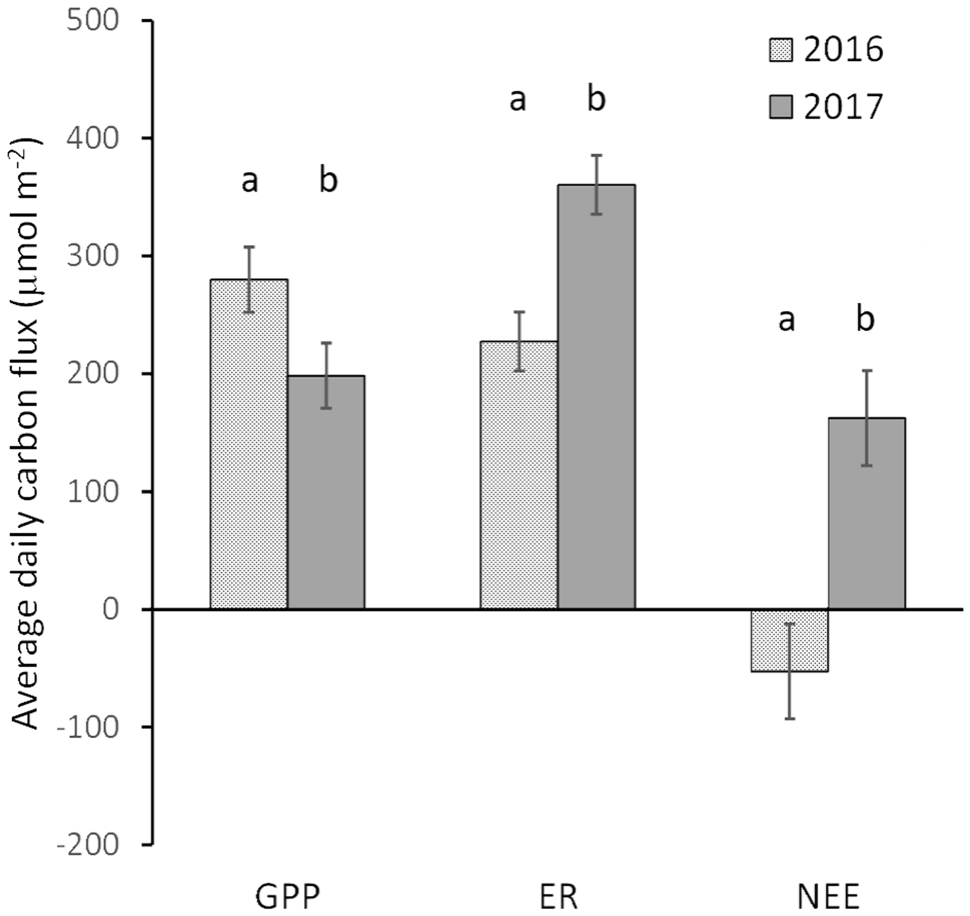
Average of daily carbon fluxes at Warra Supersite during the 2017 warm spell and the 2016 comparison period. The carbon fluxes have been partitioned into gross primary productivity (GPP), ecosystem respiration (ER) and net ecosystem exchange (NEE). 95% least significant difference (LSD) of means for each of the three component fluxes are shown. For each variable, differences between years that were statistically significant (*P* < 0.05) were indicated by different letters above the LSD bars.

**Figure 4:**
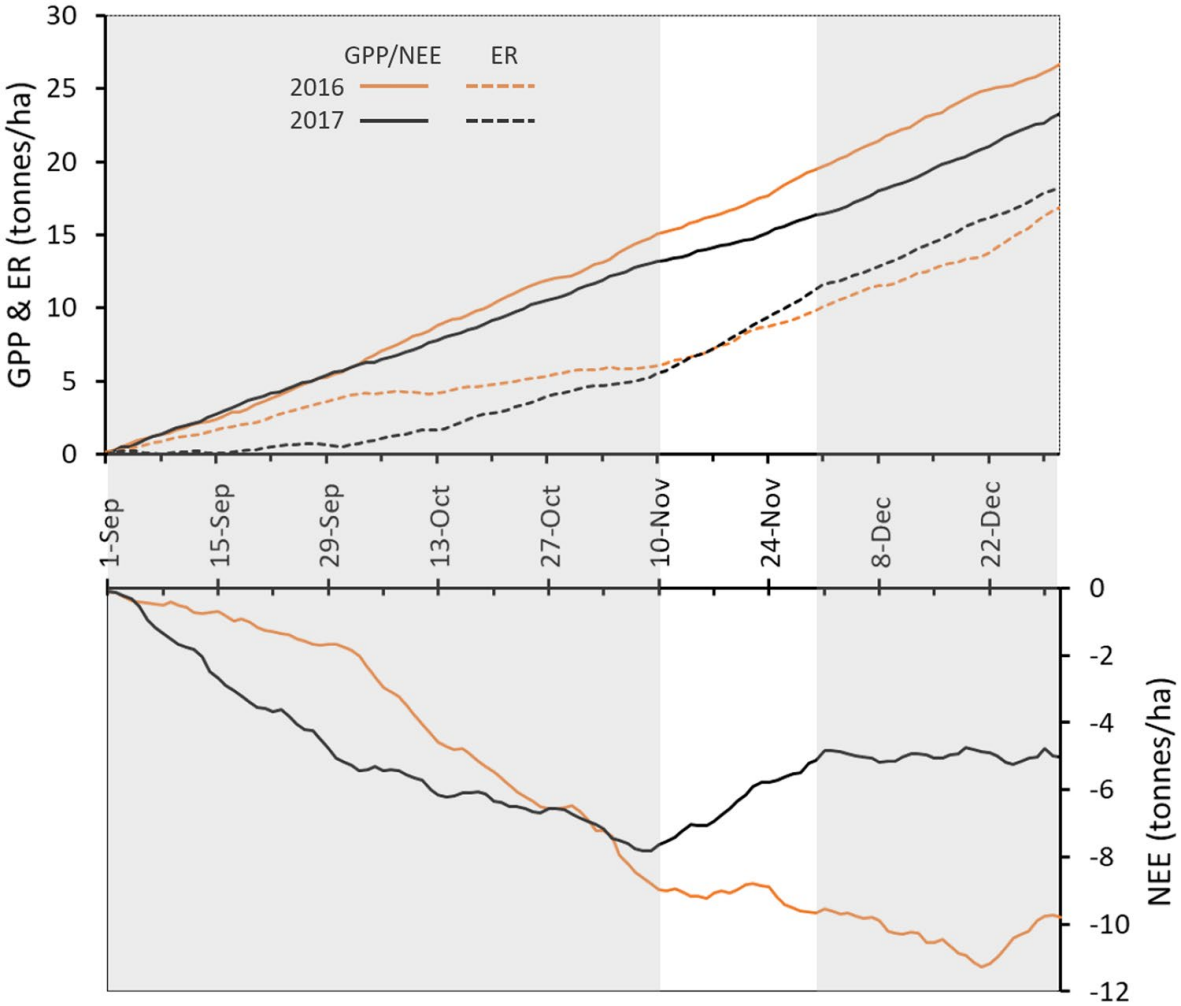
Cumulative fluxes of carbon measured at the Warra Supersite between 1 September and 31 December in 2016 and 2017. The carbon fluxes have been partitioned into gross primary productivity (GPP), ecosystem respiration (ER) and net ecosystem exchange (NEE). The 10–30 November period corresponding to the 2017 warm spell is unshaded.

Relative to the comparison period, the warm spell had significantly lower average daily gross primary productivity, GPP, (F_1,40_ = 8.9; MSE = 7894; *P* = 0.005); significantly higher average daily ecosystem respiration, ER, (F_1,40_ = 28.9; MSE = 6439; *P* < 0.001); and, significantly lower average daily net primary productivity, NPP, (F_1,40_=28.9; MSE = 16,741; *P* < 0.001) (Fig. 3). Over the duration of the warm spell, the cumulative GPP was 320 g C/m^2^—31% less than the cumulative GPP for the comparison period (Fig. 4). The cumulative ER over the 21 days of the warm spell was 582 g C/m^2^, an increase of 58% compared with the cumulative ER for the comparison period (Fig. 4). The combined effect of the lower GPP and the higher ER during the warm spell was to switch the forest at Warra from a carbon sink with an uptake of 99 g C/m^2^ over the 21 days in the comparison period to a carbon source of 261 g C/m^2^ during the warm spell (Fig. 4). This equated to a change in NEE of 360 g C/m^2^.

During the 10 weeks prior to the warm spell, GPP was significantly lower (F_1,138_ = 5.31; MSE = 6320; *P* = 0.023) than the same period in 2016, which was reflected in 12% reduction in GPP (1283 vs. 1455 g/m^2^). ER and NPP in the 10 weeks prior to the warm spell were not significantly different to the same period in 2016. In the month following the warm spell, the forest returned to being a net carbon sink—NEE returned to levels that were not significantly different from those recorded in 2016 (Kruskal–Wallis test statistic = 0.42; *P* = 0.84).

**Figure 5:**
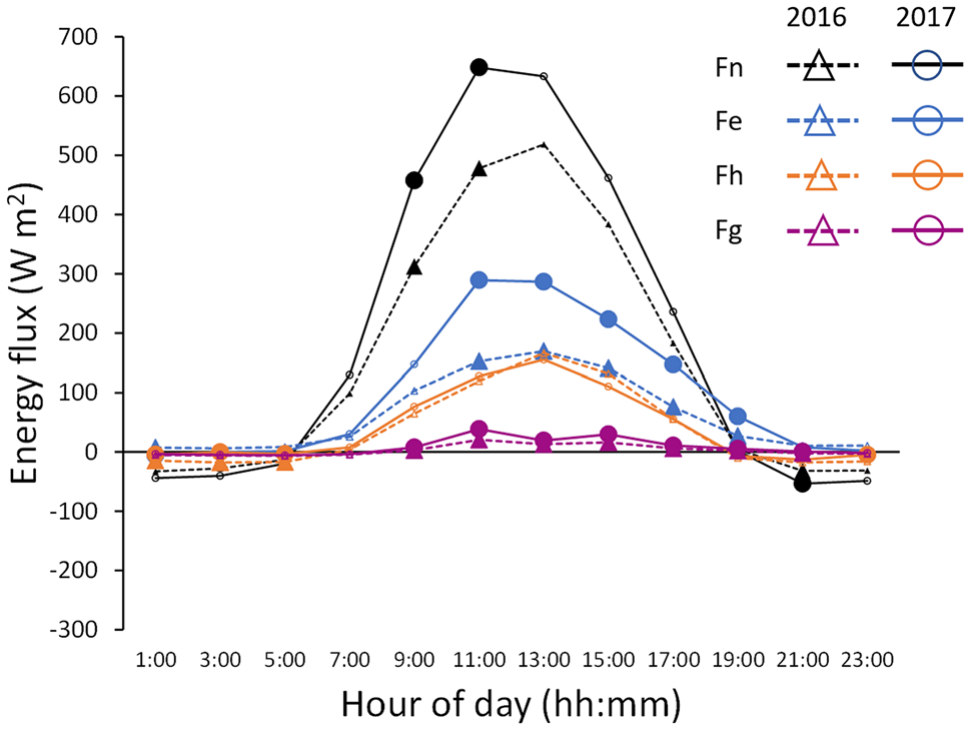
Diurnal plot of 2-hourly averages of component energy fluxes during the November 2017 warm spell and 2016 comparison period. Component fluxes that are significantly different (*P* < 0.05) between years at a time period are indicated with enlarged and filled point symbols.

The net radiation (Fn) received during the daytime hours of the warm spell exceeded that of the comparison period, particularly during the morning where the differences reached statistical significance (Fig. 5). Similarly, latent heat fluxes (Fe) during the daytime of the warm spell exceeded those of the comparison period, with those exceedances reaching statistical significance during the middle of the day and afternoon (Fig. 5). In contrast, daytime sensible heat fluxes (Fh) during the of the warm spell were comparable with the comparison period. Significant differences in Fh only occurred at night when Fh fluxes were minimal (Fig. 5). Ground heat flux was significantly higher during the daytime and evening hours of the warm spell than the comparison period, however the fluxes were small in absolute terms compared with Fn, Fe and Fh (Fig. 5). In summary, the additional net radiation received during the warm spell was dissipated through increases in latent heat rather than sensible heat. Reflecting this, the daytime Bowen ratio (Fh/Fe) was significantly lower in the warm spell than it was during the comparison period (t = 3.41, *P* < 0.001). In contrast, daytime canopy conductance during the warm spell was not significantly different from that of the comparison period (t = 0.72, *P* = 0.47).

**Figure 6:**
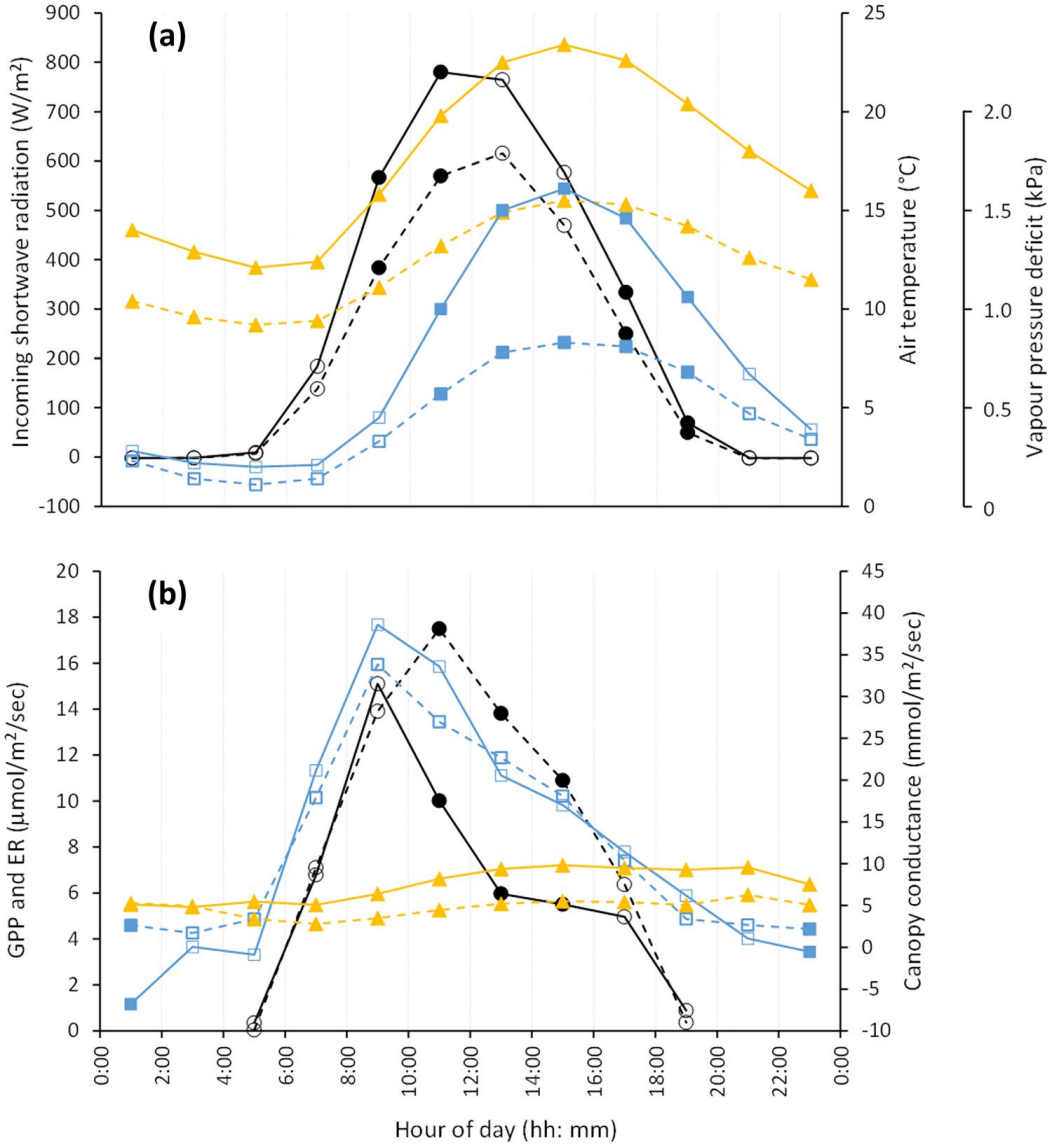
Diurnal plots of 2-hourly averages during the November 2017 warm spell (solid lines) and the 2016 comparison period (dotted lines) for: (**a**) the climatic variables of incoming shortwave radiation (black lines and circle symbols), air temperature (orange lines and triangle symbols) and vapour pressure deficit (blue lines and square symbols); and (**b**) the physiological variables of GPP (black lines and circle symbols), ER (orange lines and triangle symbols) and canopy conductance (blue lines and square symbols). Filled symbols indicate variables that are significantly different (*P* < 0.05) between years for the 2-h period shown.

For both the warm spell and comparison period, the diurnal pattern of both GPP and canopy conductance tracked incoming shortwave radiation, while ecosystem respiration tracked air temperature (Fig. 6a,b). Significantly, the diurnal pattern of canopy conductance did not correspond well with that of vapour pressure deficit, with the latter showing strong afternoon divergence between the warm spell and comparison periods that was not apparent in canopy conductance. Canopy conductance, for both the warm spell and comparison period, quickly increased during the morning, peaking mid-morning, and then slowly declined through the middle of the day and afternoon (Fig. 6b). GPP for both the warm spell and comparison period increased quickly for the first two 2-h periods in the morning. By late morning though, GPP during the warm spell dropped sharply from that of the comparison period, and that drop in GPP persisted through the afternoon (Fig. 6b). The late-morning and afternoon drop in GPP during the warm spell corresponded with average air temperatures reaching 19.8 °C or more (Fig. 6a,b).

## Discussion

Compared with same period in the preceding year, the November 2017 warm spell at Warra resulted in a reduction in GPP of 31%; an increase in ER of 58%; the forest switching from a sink to a source; a decrease in the Bowen ratio, and the maintenance of high canopy conductance. This combination of responses during such mild heatwave conditions have not been reported previously. Temperatures during the warm spell and embedded heatwaves in southern Tasmania were mild relative to those recently documented for other heatwaves affecting forest ecosystems in the southern Australian mainland^3,8,18^. This reflected the method used to determine heatwaves in this study—three consecutive days with temperatures above the 90th percentile value for that calendar period—as proposed by Perkins and Alexander^9^—rather than continuous days above an absolute temperature threshold. In this context, the warm spell was of exceptional severity, locally, and eclipsed previous records for the state by a large margin^15^. So, even using a less stringent definition of a heat-wave, the changes in carbon fluxes were large.

Eucalypt-dominated ecosystems can change from a carbon sink to carbon source during heatwaves as the result of increased ER alone, or the combination of increased ER and reduced GPP. Increased ER was the sole driver of the forest ecosystem switching from a carbon sink to a source during a heatwave in the case of *Eucalyptus* spp. at Wombat Forest during the January 2014 heatwave^18^ and *E. delegatensis* at Tumbarumba during the 2012–2013 heatwave^3^. The combined effect of increased ER and decreased GPP in causing a switch from a carbon sink to a source during heatwaves has been reported in eucalypt dry sclerophyll forests and Mediterranean woodlands in during the 2012–2013 heatwave across southern Australia^3^; in young *E. parramattensis* during an induced heatwave^7^; *E. moluccana*/*E. fibrosa* at Cumberland Plains during summer months^19^.

Declines in GPP during heatwave events in Australian eucalypt forests are believed to involve regulation of stomatal conductance to limit water loss when soils are dry^3,18^ and/or when VPDs are very high^3,7,8^. The decline in GPP during the warm spell at Warra was different. The *E. obliqua* tall forests at Warra are among the most mesic of Australia’s forests^20^ and as a result have sacrificed hydraulic safety^21^ to maintain high rates of photosynthesis across the range of leaf water potentials typically encountered^22^. Soil moisture was not limiting at Warra during the warm spell; VPDs did not reach high values and canopy conductance remained comparable with the much milder conditions during the comparison period. These moisture conditions during the warm spell when considered in the context of our knowledge of the response of photosynthesis to water potential in *E. obliqua* suggests it was unlikely stomatal regulation to limit water loss was activated during the warm spell.

GPP declines in eucalypt ecosystems that coincide with reduced latent heat flux, and attributed to low soil moisture, typically report soil water contents of less than 10%^3,18^. Soil water content at Warra did not drop below 20%. This was comparable with, or higher than, soil water content reported for heatwave events at Tumbarumba in 2012–2013^3^ and Wombat Forest in 2014^18^. High latent heat fluxes were sustained during those heatwaves at Tumbarumba and Wombat Forest as they were at Warra, but in contrast with Warra the forests showed no declines in GPP.

Declines in GPP during heatwaves in eucalypt ecosystems that are attributed to VPD-mediated reductions in stomatal conductance report VPD values that approach, or exceed, 6 kPa, typically when temperatures exceed 40 °C^3,7^. In contrast to these extreme heatwaves, conditions were much less severe during the warm spell—VPD rarely exceeded 3 kPa and the maximum VPD was only 3.3 kPa. This was also well below the average VPD (4.5 kPa) reported by Griebel et al.^18^ during the heatwave and the hottest days of summer months between 2013 and 15 at Wombat Forest where both sapflow and latent heat fluxes increased relative to baseline. Finally, the strong afternoon decline in GPP during the warm spell, which was not accompanied by reductions in canopy conductance (relative to the comparison period), is compelling evidence that VPD-driven stomatal regulation was not responsible for the reductions in GPP during the warm spell at Warra.

Estimation of canopy conductance is based on transpiration, not evapotranspiration as was done in this study. It was not possible to partition evapotranspiration into evaporation and transpiration components, although soil evaporation was likely to be small relative to transpiration based on measurements in a similar forest in Victoria^23^. Soil moisture was the main factor differing between the warm spell and the comparison period that would affect soil evaporation. The soils were drier during the warm spell compared with comparison period. Drier soils translate to a drier litter layer, based on^24^, which is the major factor in reducing soil evaporation beneath forest^25^. Accordingly, evaporation as a component of evapotranspiration, is expected to be lower during the warm spell than the comparison period. This would translate to a divergence in daytime canopy conductance, with that estimated for the comparison period declining relative to the warm spell.

Temperatures that are supra-optimal for GPP provide a simple explanation for the reduction in GPP measured at Warra during the warm spell. Bennett et al.^12^ recently documented the GPP versus temperature relationship for 17 eucalypt-dominated forest ecosystems in Australia. For each ecosystem, the GPP versus temperature relationship was a convex parabola with the vertex corresponding to the optimum temperature (T_opt_) for GPP. The T_opt_ of each ecosystem was linearly related to the historical daytime mean temperature of the ecosystem. This matched the finding of Tan et al.^11^ for tropical rainforests. Bennett, et al.^12^ found the temperature optimum for GPP at Warra was 16 °C—one of the lowest for the sites they examined. This reflected the relatively cool climate in southern Tasmania. Nearly 75% of the 30-min average temperature records during daylight hours of the warm spell were in the supra-optimal range. Bennett et al.^12^ also found that for Warra, together with tropical rainforest sites, GPP showed a high temperature sensitivity (rate of decline in GPP as temperature departs from the optimum) compared with other eucalypt forests in Australia. The causes of this higher temperature sensitivity at Warra have not yet been determined. Many of the other temperate eucalypt sites examined by Bennett et al. were water-limited, particularly during heatwave events as reported by^3^ so that declines in GPP at higher temperatures are likely mediated through diminished canopy conductance (inferred from very low latent heat fluxes) rather than temperature, directly.

Based on the evidence presented, the *E. obliqua* tall forest at Warra switched from a strong carbon sink to a source during the 2017 warm spell because daytime temperatures exceeded the site optimum temperature for GPP for most of the three-week period. The reduction in GPP and the increase in ER during the warm spell indicate their decoupling as described by Duffy et al.^13^. The antecedent conditions in 2017 resulting in reduced GPP (compared with the same period in 2016) but not ER, is also consistent with^12^ and can be explained by a higher frequency of daytime temperature extremes in 2017. Both extremes of temperature would cause reductions in GPP but the reduced ER at low temperatures would cancel the increased ER at the high temperatures. The forest was resilient to the warm spell and quickly recovered to being a carbon sink once temperatures moderated in the subsequent month. Such resilience to transient heatwave events has been seen in other forests^26^, but whether such recovery confers longer-term advantages for coping with warmer temperatures, through acclimation, is difficult to predict. Evidence is conflicting with some studies showing acclimation to warmer temperatures^27–30^, others do not^31,32^. Recent studies have shown the forests at Warra showed an atypical lack of seasonal temperature plasticity for photosynthesis^33^ and respiration^34^ suggesting acclimation to warmer temperatures may be limited. Further, there is some evidence that acclimation to warmer temperatures does not confer greater tolerance to future heatwaves^35^.

Heatwave events will become more regular as temperatures increase, which may lead to the some existing ecosystems becoming long-lasting carbon sources^13^. Persistent stagnation of growth as forests cross tipping points of heatwave recurrence are starting to be reported^36^. It is possible the tall eucalypt forests of southern Tasmania have reached tipping points from warming temperatures in the past. A syndrome called “regrowth dieback” expressed as stagnating growth and progressive dieback and eventual mortality of mid-aged and mature eucalypt forest in Tasmania during the 1960–1980s was attributed to drought events. However, key events such as commencement of declining growth rates linked to developing dieback symptoms in 1959^37^ and an intensification of regrowth dieback in 1972–1973^38^ were also warm years embedded within a longer period of much warmer growing season temperatures shown in temperature records from the Bureau of Meteorology’s Cape Bruny Lighthouse station. It may be worthwhile revisiting forest inventory data that was collected from tall eucalypt forests in southern Tasmania during the 1950–1980 s and examining the development and intensification of regrowth dieback through the lens of higher temperatures.

Based on the historical climate of southern Tasmania the 2017 warm spell was an exceedingly rare event. Such events, however, will become more common as temperatures continue to increase and may result in Tasmania’s existing tall eucalypt forests transitioning to become carbon sources in the future. The tall eucalypt forests in Tasmania are among the most productive natural forest ecosystems globally and can store large amounts of carbon^39–42^. There would be many adverse consequences if these forests were to transition to long-lasting carbon sources with warming temperatures, particularly if understorey species such as *Nothofagus cunninghamii* supplant eucalypts to become the canopy-species. This has occurred previously in some eucalypt-dominated ecosystems in Tasmania^43,44^ with the resultant forests having substantially lower carbon-carrying capacity^45^. While adaptation options to reduce the risks of adverse effects of climate change have been evaluated for tall eucalypt forests^46^, the direct effect of higher temperatures were not considered. Our understanding of the direct effect of warmer temperatures on the productivity and ecological processes in tall eucalypt forests is currently rudimentary. This will need to change, and quickly.

## Methods

### Site description

Warra Supersite, (Lat: 43° 5′ 42ʺ S; Long: 146° 39′ 16ʺ E) is located on a floodplain of the Huon River within the Warra Long Term Ecological Research site (https://warra.com/) 60 km southwest of Hobart, Tasmania. The forest at the Supersite is a *Eucalyptus obliqua* tall forest with a canopy height of 50–55 m, overtopping a 15–40 m tall secondary layer of rainforest and wet sclerophyll tree species. Ferns dominate the ground layer. The forest is very productive with an aboveground biomass of 790 tonnes/ha^16^ and a leaf area index of 5.7 m^2^/m^2^^47^.

The Supersite is within the Tasmanian Wilderness World Heritage Area (TWWHA). That part of the TWWHA experiences infrequent, but sometimes intense, wildfire. Except for a small proportion of mature (> 250 years-old) *E. obliqua* trees, the current forest resulted from seedling regeneration following the last major wildfire in that part of the landscape in 1898. No timber harvesting has ever been done in the forest at the Supersite.

The climate at Warra is classified as temperate, with no dry season and a mild summer^48^. Mean annual rainfall measured at the nearby Warra Climate Station (Bureau of Meteorology Station 097024) is 1736 mm and the mean daily temperature is 14 °C and 5.6 °C in January and July, respectively. The soil at the site is a Kurosolic Redoxic Hydrosol^16^.

### Analysis of historical heatwaves in southern Tasmania

Daily maximum temperature records from the Bureau of Meteorology station at Cape Bruny Lighthouse (station number 94010) were extracted from the Bureau of Meteorology’s online climate data portal (http://www.bom.gov.au/climate/data). Cape Bruny Lighthouse is one of the 112 stations in the ACORN-SAT network of Australia’s reference sites for monitoring climate change^49^. The station provides a record of daily maximum temperature measurements commencing in 1923 and spanning almost a century. It is the southern-most station in the ACORN-SAT network; is 59 km south-east of the Warra Flux Site; and bounds the south-eastern extent of *E. obliqua* tall forest in Tasmania.

Missing temperature measurements represented less than 0.6% of the 35,795 records collected at Cape Bruny Lighthouse between January 1st 1923 and December 31st 2020. The missing measurements were gap-filled using predicted values calculated from linear regression models constructed from measurements made at nearby Bureau of Meteorology stations (listed in order of proximity to Cape Bruny Lighthouse and priority for gap-filling)—Cape Bruny Automatic Weather Station (1997-present), Hastings Chalet (1947–1987) and Hobart-Ellerslie Road (1892-present).

Average, standard deviation and 90th percentiles of daily maximum temperature were calculated for each calendar day of the year. Further analysis of heatwaves was restricted to the period between the beginning of August and the end of February. This period bounds the growing season of the forest at the Warra Supersite when there is normally a net carbon gain by the forest (Wardlaw unpublished data). Heatwaves were identified as three or more consecutive days with maximum temperatures that met or exceeded the 90th percentile value sensu Perkins and Alexander^9^. For each heatwave event that was identified, the following three statistics were calculated: (1) average daily maximum temperature during the heatwave; (2) summed departures (as standard deviations) from average daily maximum temperature during the heatwave; (3) summed departures (as standard deviations) from average daily maximum temperature of the 21 day period centred on the middle day of the heatwave. The November 2017 heatwave, as described by these three statistics, was ranked against all the other heatwave events identified between 1923 and 2020 at Cape Bruny Lighthouse. In addition, the z-score was calculated to measure the magnitude of the departure of the average daily maximum temperatures during the November 2017 heatwave from the long-term average of this 21-day period. Those statistics were also calculated for the same period in 2016.

### Weather conditions at Warra Supersite during the 2017 warm spell

Four attributes of weather were used to describe the November 2017 warm spell—air temperature, vapor pressure deficit (calculated from temperature and relative humidity), incoming shortwave radiation and soil moisture. Air temperature and relative humidity were measured using an HMP155A probe (Vaisala, Finland) and incoming shortwave radiation was measured using a CNR4 radiometer (Kipp and Zonen, The Netherlands). Both instruments were mounted 80-m above ground level at the top of the Warra Flux tower. Data was processed to 30-min averages and logged onto a CR3000 datalogger (Campbell Scientific, Logan, USA).

Soil moisture was measured by time-domain reflectometry using two CS616 soil moisture probes (Campbell Scientific, Logan, USA) each installed at a depth of 20 cm. These probes were installed in two pits approximately 40 m west of the tower. Soil moisture data were processed to 30-min averages and logged onto a CR1000 datalogger (Campbell Scientific, Logan USA).

### Turbulent fluxes at Warra Supersite during the November 2017 warm spell

Measurement of turbulent fluxes (carbon, water and energy) were done by eddy covariance (EC) using a closed-path infra-red gas analyser (Model EC155, Campbell Scientific Inc., Logan, USA) to measure CO_2_ and H_2_O concentrations and a 3-D sonic anemometer (Model CSAT3A, Campbell Scientific Inc, Logan, USA) to measure turbulent wind vectors and virtual air temperature. The sonic anemometer and infra-red gas analyser were mounted at 80-m above the ground at the top of the Warra Flux tower. Storage of CO_2_ and H_2_O beneath the forest canopy was measured by a profile system (Model AP200, Campbell Scientific Inc, Logan, USA ), with sampling heights of 2, 4, 8, 16, 30, 42, 54, 70 m. Temperature sensors in aspirated shields (Model 110-ST, Apogee Instruments, Logan, USA) were co-located with the CO_2_/H_2_O sample inlets of the profile system. High frequency (10 Hz) measurements of turbulent fluxes were processed to 30-min averages in a datalogger (Model CR3000, Campbell Scientific, Logan USA). High frequency (2 Hz) of CO_2_ and water concentration measurements were processed to 15-s averages sequentially for each profile sample height in a datalogger (Model CR1000, Campbell Scientific, Logan, USA). Thus, each inlet was sampled for a 15 s interval every 2 min. The rate at which sub-canopy storage of CO_2_ changed was calculated from changes in the quasi-instantaneous (2-min) vertical profile concentrations beneath the tower at the beginning and end of each 30-min flux averaging period using the method of McHugh^50^.

Soil heat flux (SHF) was measured to enable calculation of energy balance that was needed to partition energy fluxes into latent and sensible heat. SHF was measured using five SHF plates (Model HFP01SC, Hukseflux, Delft, The Netherlands) inserted in the soil at depth 8 cm adjacent to the two pits in which the soil moisture probes were installed. Each of the five SHF plates were allocated to one of the two soil pits in a 2–3 split. Changes in soil temperature was measured by an averaging thermocouple (Model TCAV, Campbell Scientific Inc, Logan, USA) inserted into the soil above each SHF plate at depths of 2 and 6 cm. Soil moisture measurements at 20 cm depth were as described previously. Heat flux, soil temperature and soil moisture data were processed to 30-min averages on a datalogger (Model CR1000, Campbell Scientific Inc, Logan, USA).

Raw 30-min flux, CO_2_ storage and climate data were processed by the standard OzFlux QA/QC processing stream^51^ using PyFluxPro Version 1.0.2 software. Fluxes (carbon and energy) adjusted for storage were computed at the mid-stage (level 3). At the final stage of data processing (level 6), gap-filled net ecosystem exchange (NEE) data were partitioned into gross primary productivity (GPP) and ecosystem respiration (ER) using the u*-filtered night-time CO_2_ flux records to calculate ER with the SOLO artificial neural network algorithm as described in^51^. The standard conventions of the global flux network were adopted in partitioning NEE as described in^52^.

The full period between 10 and 30th November 2017 was defined as the November 2017 warm spell. The climate and fluxes measured during this period were compared with measurements of those made during the same calendar days of the preceding year, 2016. The carbon fluxes measured in the 10 weeks before (1 September–9 November) and the month after (1–31 December) the 2017 warm spell period were also compared with the same periods in 2016. This was done to ascertain whether changes in carbon fluxes during the 2017 warm spell we not due to differences in antecedent weather conditions and, whether or not differences in carbon fluxes arising from the warm spell persisted after the warm spell.

### Data analysis

For each day of the 10–30 November period, daily sums were calculated for measurements of carbon fluxes and incoming shortwave radiation (Fsd), while daily averages were calculated for air temperature, VPD and soil moisture. Quantile plots, done for Ta and VPD, used 30-min data during daytime hours (when Fsd > 0). The significance of differences in measurements during the 10–30 November period among the two years of each variable were tested by analysis of variance. Tests were first done to confirm the data for each variable were normally distributed and between-group variances were homoscedastic. Log-transformation was used to correct skewness in the VPD data. Soil moisture data were strongly skewed, and transformation was unable to correct. For this variable, the Kruskal–Wallis method was used to test the significance of differences in medians among the two years. These analyses were repeated for the 10 weeks (1st September–9th November) leading up to the warm spell and the 4 weeks (1st–31st December) following the warm spell to examine antecedent conditions and subsequent recovery from the warm spell, respectively.

The energy fluxes were examined for evidence of coupling between GPP, transpiration and canopy conductance. Closure of the energy balance was first determined for the two periods to ensure comparability of the energy fluxes for the 2017 warm spell period and the corresponding period in 2016. This was done by firstly resampling the 30-min data and calculating 2-hourly averages of latent heat flux (Fe), sensible heat flux (Fh), net radiation (Fn) and ground heat flux (Fg), then fitting linear regressions of Fe + Fh against Fn–Fg for dates encompassing the warm spell in each of two years. Peak energy storage of the biomass, Fb, in the forest at Warra was estimated as 40 W m^−2^ using the method described in^17^. That estimate used the value of LAI of 5.72 based on the average of periodic measurements of LAI at Warra reported in^47^ and the value of 22.0 for the quadratic mean radius at breast height (1.3 m) calculated from tree measurements in a 1-ha plot adjacent to the Warra Flux tower (detailed in^47^). The ratio of energy storage in the biomass and ground heat flux at their respective daily maxima was calculated, assuming their respective diurnal peaks coincided. This ratio was then applied to each 2-h average of ground heat flux measured in the warm spell period in 2017 and the corresponding period in 2016. Available energy was recalculated using the formula Fn–(Fg + Fb). Analysis of variance was used to test the significance of differences between the 2017 warm spell and the corresponding period in 2016 of each component energy fluxes (Fn, Fe, Fh and Fg) for each of the twelve, 2-h periods, in the day. Kruskal–Wallis rank test was used if a variable had a non-normal distribution or exhibited heteroscedasticity. The Bowen ratio, which is the ratio between Fh and Fe, was calculated for each 2-h period during daytime hours. The 2-h average data were non-normal and heteroscedastic so testing the significance of differences in daytime Bowen ratio between the warm spell and comparison period was done using 2-sample t-test with unequal variance.

Latent heat flux was converted to evapotranspiration by dividing the measured latent heat flux by the latent heat of vaporisation of water. Evapotranspiration was used as a proxy of transpiration on the assumption that evaporation was a minor component of evapotranspiration in the tall *E. obliqua* forest at Warra based on measurements of soil and litter evaporation in similar forests by^23^. An estimate of total canopy conductance of sunlit leaves, G_t_, was calculated from transpiration (E) and vapor pressure deficit, VPD, using the Skelton et al.^53^ adaptation of the method developed by Hogg and Hurdle^54^, whereby:

$${\text{G}}_{{\text{t}}} = (\upalpha /1000){\text{E}}/{\text{VPD}}$$

The atmospheric pressure of water vapor, α, is equivalent to ρ_w_G_v_T_k_, where ρ_w_ is the density of water (*c* 1000 kg m^−3^), G_v_ is the universal gas constant for water vapor (0.462 m^3^ kPa kg^−1^ K^−1^) and T_k_ is air temperature (in K = Ta + 273.15). G_t_ (in mmol m^−2^ s^−1^) was calculated for each 2-h period during the 2017 warm spell and the same calendar days in 2016 using each period’s corresponding values of E, VPD and Ta. Records were excluded if rain fell during the 2-h period. The significance of differences in daytime canopy conductance between the 2017 warm spell and the 2016 comparison period was tested using a two-sample t-test as the data were strongly skewed.

The diurnal patterns of GPP, ER and canopy conductance were compared with incoming shortwave radiation, air temperature and vapour pressure deficit. Each 30-min record of the six variables was recoded to its corresponding 2-h time interval. Analysis of variance was used to test for significance of differences between the warm spell and comparison period for each of the twelve 2-h diurnal periods of the six variables. Kruskal–Wallis rank test was used in the data were non-normal or heteroscedastic. Time series plots of diurnal 2-hourly averages for each of the six variables were plotted and visually compared.

## Data Availability

Climate data were accessed from the online repository of the Bureau of Meteorology (http://www.bom.gov.au/climate/data/?ref=ftr) for the stations referenced: Cape Bruny Lighthouse (094010), Cape Bruny Automatic Weather Station (094198), Hastings Chalet (094027), Hobart—Elderslie Road (094029). Weather data, turbulent fluxes and partitioned carbon fluxes for Warra Supersite for the 10–30 November period in 2015, 2016 and 2017 are available on the OzFlux Data Portal (http://data.ozflux.org.au/portal/home.jspx).
